# Optical techniques for cervical neoplasia detection

**DOI:** 10.3762/bjnano.8.186

**Published:** 2017-09-06

**Authors:** Tatiana Novikova

**Affiliations:** 1 LPICM, CNRS, Ecole polytechnique, University Paris Saclay, Palaiseau, France

**Keywords:** cervical intraepithelial neoplasia, confocal endomicroscopy, Mueller polarimetry, nanotheranostics, optical coherence tomography, optical spectroscopy, Raman spectroscopy

## Abstract

This paper provides an overview of the current research in the field of optical techniques for cervical neoplasia detection and covers a wide range of the existing and emerging technologies. Using colposcopy, a visual inspection of the uterine cervix with a colposcope (a binocular microscope with 3- to 15-fold magnification), has proven to be an efficient approach for the detection of invasive cancer. Nevertheless, the development of a reliable and cost-effective technique for the identification of precancerous lesions, confined to the epithelium (cervical intraepithelial neoplasia) still remains a challenging problem. It is known that even at early stages the neoplastic transformations of cervical tissue induce complex changes and modify both structural and biochemical properties of tissues. The different methods, including spectroscopic (diffuse reflectance spectroscopy, induced fluorescence and autofluorescence spectroscopy, Raman spectroscopy) and imaging techniques (confocal microscopy, optical coherence tomography, Mueller matrix imaging polarimetry, photoacoustic imaging), probe different tissue properties that may serve as optical biomarkers for diagnosis. Both the advantages and drawbacks of these techniques for the diagnosis of cervical precancerous lesions are discussed and compared.

## Review

### Introduction

**Figure 1:**
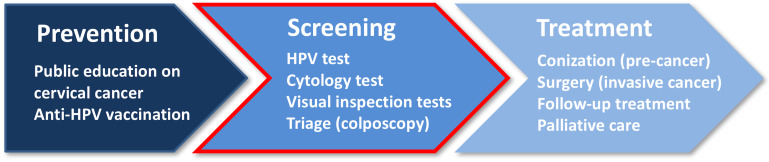
Key steps for addressing cervical cancer as public health concern. New optical technologies and innovative approaches for the improvement of early detection of cervical pre-cancer (second step) are discussed in this paper.

Cervical cancer remains one of the major health issues, causing 266000 deaths of women worldwide in 2012 [1]. While the highest incidence rate of cervical cancers (approximately 70%) is observed in developed countries, the cervical cancer mortality rate is highest in low-income countries, where the regular screening by Papanicolaou (Pap) test, colposcopy, biopsy and curative treatment are not routinely available because of lack of health infrastructure, trained practitioners and necessary resources [2]. The high mortality rate of cervical cancer may be reduced by implementing the integrated strategy which includes the prevention, screening and treatment of the disease [3] (Figure 1).

**Figure 2:**
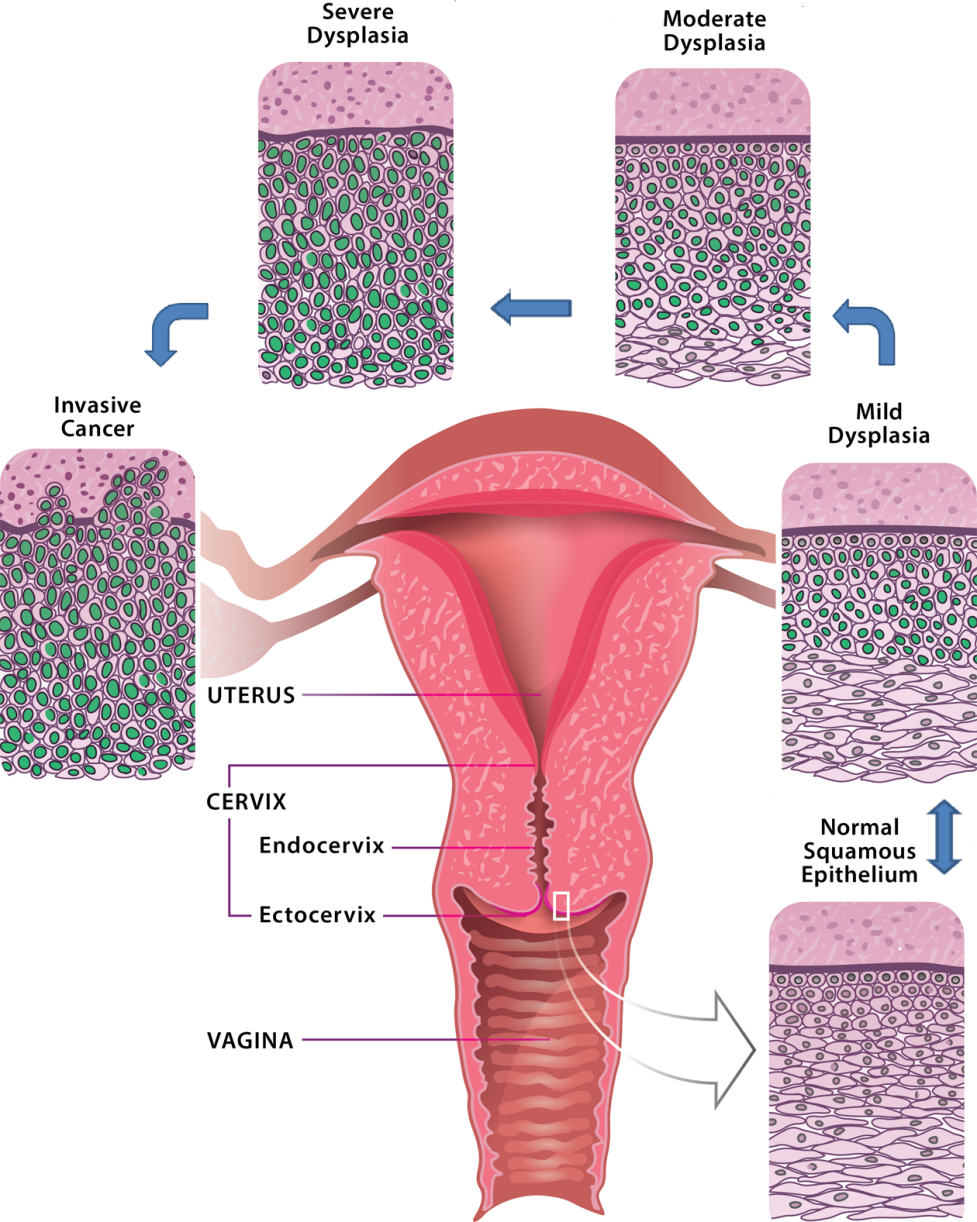
Cross-section of uterus and vagina; schematics of cervical intraepithelial neoplasia development.

There is conclusive evidence that the majority of cervical cancer cases (95–98%) is caused by the infection with cancerogenic strains of *human papillomavirus* (HPV) [4–6]. Most of these infections are cleared by the immune system within one to two years. If carcinogenic HPV infection is not cleared, the virus invades the cells at the junction of squamous epithelium of the ectocervix and columnar epithelium of endocervical canal (cervical squamocolumnar junction CSJ) [7–9]. The location of the squamocolumnar junction relative to the external orifice, or external os (cervix opening to the vagina, see Figure 2) shifts over the lifetime of a woman.

The transformation zone, defined as an area limited by the positions of original and the active squamocolumnar junctions is most susceptible to HPV infection. When HPV gets a foothold and reproduces itself, it can invade the cells of the basal layer, which separates epithelium from underlying connective tissue and, eventually, rise to the epithelium surface with the mature squamous cells. The virus infection gradually induces severe damage. HPV-infected cells may become malignant if the virus inserts its cancer-causing genes into the DNA of the host cell.

The staging of the disease is based on morphological criteria and tissue architecture, namely, on the thickness of the involved epithelium layer (Figure 2). When one third of the epithelium is affected by disorganized growth and cytological atypia we talk about mild dysplasia or cervical intraepithelial neoplasia (CIN) of 1st grade (CIN 1). Such abnormality can regress and disappear on its own. Moderate (CIN 2) dysplasia involves two thirds of the epithelium, while severe dysplasia (CIN 3) spans over the whole epithelium depth. At this stage, it is already highly unlikely that precancerous epithelial lesions will clear spontaneously. According to the Bethesda system [10], the low-grade squamous intraepithelial lesion (LSIL) usually indicates mild dysplasia (CIN 1), high-grade squamous intraepithelial lesion (HSIL) refers to moderate and severe dysplasia (CIN 2–3). This classification system is used for reporting cervical cytological diagnostics and for choosing different treatment strategies for each group.

Left untreated, severe dysplasia will grow and break a basal membrane and eventually evolve into an invasive cancer. This process is very slow and may take over ten years after the infection. It makes cervical cancer perfectly suited for the effective management by screening according to criteria defined by the World Health Organization [11–12].

Recent discovery and subsequent mass use of the vaccines against HPV hold promise for the prevention of cervical cancer and will significantly improve the situation at large [13]. Those vaccines, however, need to be applied early in life, and cannot cure already existing conditions. Furthermore, none of those vaccines create complete immunity against all HPV types, and the price of these vaccines remains quite high. So, improvements in the management of HPV infection are still needed, especially for the population in low-income countries.

In high-resource settings a regular screening by the cytopathological Pap test is performed for an early detection and prevention of cervical cancer. Cells collected from the external os of the cervix are studied under a microscope. If abnormal cells are detected, further diagnostic testing in the form of colposcopy is often recommended for the localization and marking out of metaplasia.

The visual examination of the cervix for metaplastic lesions with a colposcope is done after the application of acetic acid and then repeated after the application of iodine Lugol’s solution (both work as contrast enhancing agents). Normally, the biopsies (removal of a small tissue sample for examination by a pathologist) are taken from the areas whitened by acetic acid and those which are not colored by iodine. If the analysis of histological cuts by pathologists ultimately confirms the presence of a high grade malignant lesion (CIN 2–3), the abnormal zone is surgically removed by cervical conization. This is a minimally invasive curative treatment which can completely eliminate the disease provided it was diagnosed at an early stage before the transformation into an invasive cancer. This treatment has minimal adverse effect on fertility and reproductive functions of women.

It is worth to mention that the results of colposcopy may also be affected by the presence of non-neoplastic cervical diseases and demographic factors such as age and parity. Thus, the accuracy of colposcopy strongly depends on the level of training and experience of clinicians performing the test. As stand-alone diagnostic method colposcopy has a quite high sensitivity (ratio of true positive over the sum of true positive and false negative diagnosis) of over 90% in detecting HSIL and cancer (CIN 2+). But the specificity (ratio of true negative over the sum of true negative and false positive diagnosis) of colposcopy for the detection of CIN 2–3 is reported to be relatively low (23–87%) [14–21]. Even if the diagnosis of a CIN 2–3 lesion is confirmed by histological analysis, an additional difficulty is the correct delimitation (“mapping”) of the neoplasia zone for complete treatment. This problem arises because of the lack of contrast between healthy and neoplastic zones of the cervix in colposcopy images viewed by surgeon-gynecologists. Because of these drawbacks of conventional colposcopy there is an ongoing research and exploration of different optical techniques (spectral or imaging, wide-field or scanning) for the accurate detection of cervical neoplasia.

**Figure 3:**
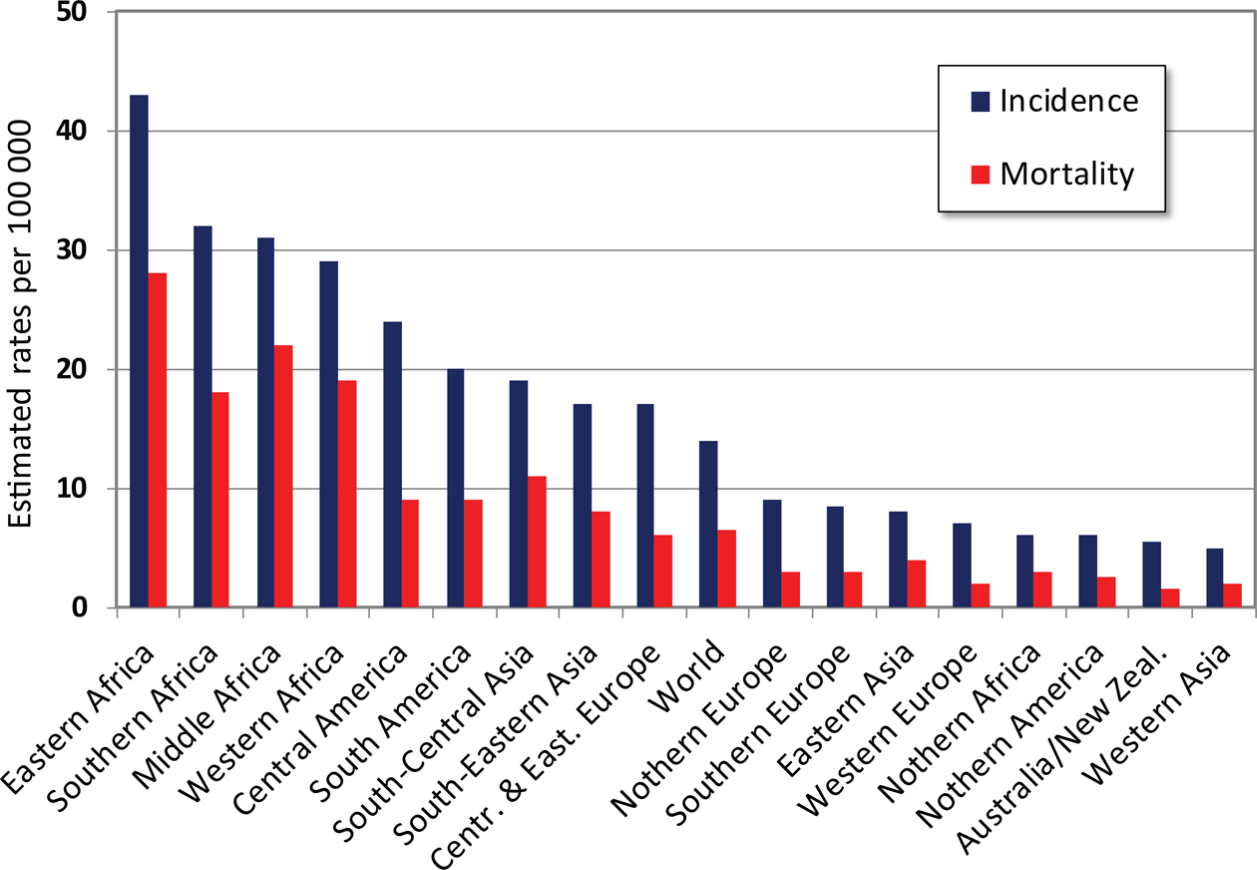
Cervical cancer estimated incidence, mortality and prevalence worldwide in 2012. Adapted from [22].

Current management of cervical cancer (implementation of screening, anti-HPV vaccination and treatment programs) has significantly decreased the mortality rate in highly developed countries during last decades. At the same time the incidence and mortality rates in the middle and low-income countries did not improve and remain significantly high due to insufficient awareness about cervical cancer among women and health providers, lack of access to HPV vaccination, absence of screening and treatment programs. This puts women at the increased risk of developing invasive cervical cancer (Figure 3).

In current programs for screening and diagnosis of cervical cancer the critical issue is an increase of efficiency and accuracy of screening and diagnostics techniques. Typically it requires up to three visits to a medical professional and several weeks in total in order to obtain the diagnosis and treatment, if necessary [23]. The implementation of new optical techniques may bring an alternative to the Pap/HPV test for screening and an improvement of colposcopy for guiding the biopsy and diagnosis. The performance of new techniques is estimated in terms of accuracy, time and cost of diagnostics, combined with patient comfort, which is relevant to the rate of participation in screening programs.

Currently several optical methods such as diffuse reflectance spectroscopy, fluorescence spectroscopy, Raman spectroscopy, in vivo confocal microscopy, optical coherence tomography and multi-wavelength imaging Mueller polarimetry, as well as the combination of different techniques have been explored to improve the detection of cervical neoplasia. The results of these studies as well as current trends to miniaturization of diagnostic instruments will be discussed further.

### Optical spectroscopy and imaging

**Figure 4:**
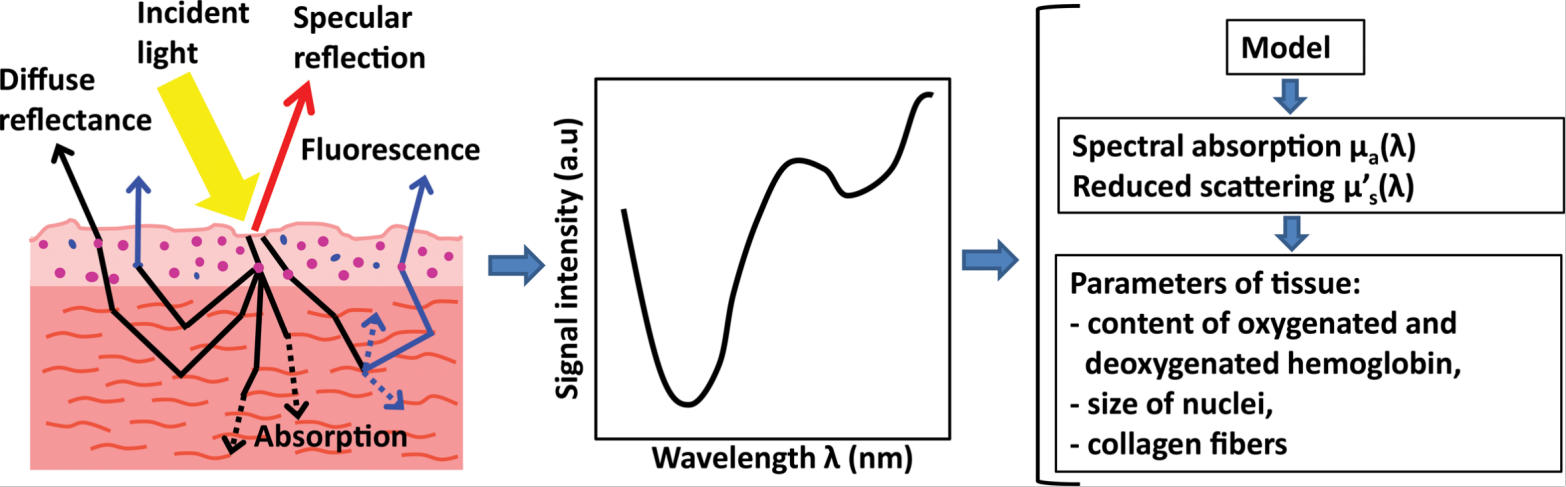
Diffuse reflectance and/or fluorescence spectroscopy for the optical analysis of tissue; λ is a wavelength. Adapted from [34].

In vivo diffuse reflectance optical spectroscopy (DRS) exploits the fact that abnormal zones of the cervical epithelium illuminated with a low-power broadband light source produce different backscattering spectra compared to normal cervical tissue in the visible wavelength range. Such difference in spectra detected by an optical sensor can be used in order to identify neoplastic lesions of the cervical epithelium. DRS is an indirect optical technique and may require either fitting of measured spectra with multi-parametric models describing the realistic optical properties of tissue [24] or using an efficient classification algorithm of optical spectra for the detection of HSIL [25–26]. The propagation of light in a scattering medium is usually modeled by the Monte Carlo algorithm. The fit of the measured spectral data with the optical model of tissue provides the effective values of diagnostically relevant model parameters, e.g., reduced scattering coefficient and absorption coefficient. In the optical model of tissue these parameters are linked to the size and density of the scatterers, total hemoglobin (Hb) concentration and Hb saturation with oxygen, which can be used as optical markers to assess and grade CIN lesion. The principle of using diffuse reflectance and fluorescence spectroscopy for tissue diagnostics is illustrated in Figure 4.

It has been demonstrated that the total concentration of Hb, which is responsible for absorption in tissue in the visible wavelength range, was statistically higher in CIN 2–3 compared to normal cervical tissue [24,27]. This effect was attributed to an increased density of micro-vessels in the stroma of neoplastic tissue and stromal angiogenesis [28–30]. The observed drop in scattering in CIN 1–3 zones was attributed to the degradation of the stromal collagen matrix of the cervix related to both decomposition of collagen fibers and decrease in concentration of collagen cross-links [31–32].

Despite the observed common trends for DRS optical markers with the evolution of CIN lesions there is a significant variability of parameter values in different patients depending on their age as well as presence of non-neoplastic lesions [33]. The shortcomings of DRS as a tool for screening and diagnosis are related to the fact that the estimation of optical parameters may be degraded by both correlation of model parameters and instrument-dependent response. It increases the uncertainty of threshold parameter values used for diagnostics and choice of treatment strategy, when either watchful waiting accompanied by HPV/Pap tests or active treatment is further needed [34–38]. The use of spectra classification algorithms (e.g., Bayesian variable selection, neural networks, library approach, multivariate statistical analysis) may bring its own set of the problems: high-dimensionality of data, insufficient number of data for training, overtraining because of too many tuning parameters [25]. Moreover, Mirkovic et al. [39] reported that even in healthy cervical tissue a transformation zone (area of most probable location of HSILs) and squamous epithelium are spectroscopically different because of their anatomical differences. This effect can also have impact on the diagnostic parameters extracted from the spectroscopic measurements. Using optical spectroscopy as a complementary technique to colposcopy aims to examine the patients with inconclusive Pap test cytological results and to guide the biopsies [25,40].

**Figure 5:**
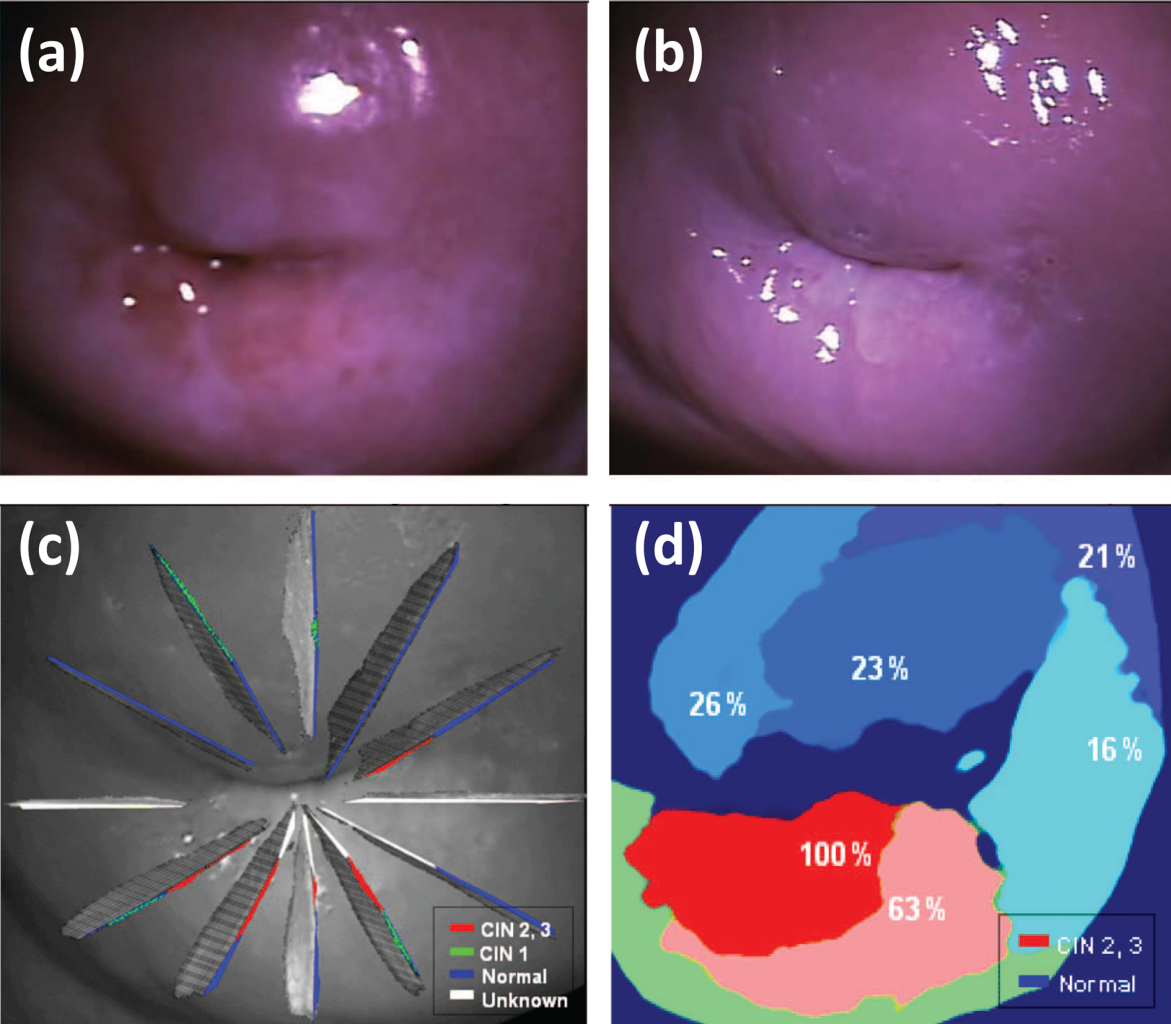
Reflectance colposcopic images (a) before and (b) after application of acetic acid; (c) reconstructed histological map of lesions CIN 1, 2, and 3; (d) diagnostic map of disease probability provided by an automated multi-classifier. Reproduced with permission from [41], copyright 2008 Society of Photo-optical Instrumentation Engineers.

Point-probe optical spectroscopic instruments may also be used for scanning the suspicious sites of the cervix. However, this approach is laborious and time-consuming and the possibility to miss the potential lesions is not negligible. Hence, these techniques are not suitable for CIN screening in real settings. The instruments that perform a multi-spectral wide-field imaging of the whole cervix are required to address these issues. Park et al. [41] developed an algorithm for the automated analysis of colposcopic images taken with a multispectral digital colposcope before (Figure 5a) and after (Figure 5b) application of acetic acid. They explored the ratio between the reflected intensities of green and red light and the changes in the reflectance images induced by acetic acid as optical markers for differentiating HSIL and cancer from LSIL and healthy cervical tissue. In their study of 29 patients a sensitivity of 79% and a specificity of 88% for HSIL detection were reported using histological analysis of excised cone biopsies (Figure 5c) as the gold-standard diagnosis technique.

The advanced version of the automated domain-specific image analysis algorithm for the detection of cervical precancerous lesions identified first the regions of squamous and columnar epithelium [42]. Transformation zone and external os were delimited on the image taken before the application of acetic acid using color and texture information. Domain-specific anatomical features related to tissue types were integrated in the conditional random field probabilistic model for the segmentation of images taken after the application of acetic acid. The clinical data from 48 patients were examined with the proposed image analysis algorithm resulting in an average sensitivity of 70% and specificity of 80% in detecting neoplastic areas, when using histopathology analysis as gold-standard diagnosis. Lower average sensitivity compared to conventional colposcopy performance was attributed to the fact that during the patient-based colposcopy analysis a delimitation of abnormal zones in images was not carried out.

### Fluorescence spectroscopy and imaging

While the scattering of light by biological tissue plays the main role in DRS, the absorption and emission of light by matter are the key steps in fluorescence spectroscopy. The use of fluorescence spectroscopy for the screening and diagnosis of cancer is related to the ability of this technique to probe the molecular composition of tissue and observe the distribution of specific molecules. When light of a chosen excitation wavelength illuminates the sample, the tissue molecules are exposed to light having an energy that may match a possible electronic transition within the molecule. Consequently, part of incident radiation will be absorbed as the electron is lifted to a higher energy orbital. During de-excitation (return of electron to the ground state) those molecules release energy in the form of light of a specific emission wavelength (usually different from the excitation wavelength), which can be measured by a detector. The fluorescence signal is a superposition of various emission signals of different wavelengths and intensities. It depends on the excitation wavelength and on the presence and concentration of fluorophore molecules in the tissue.

Depending on the type of investigated fluorophores (endogenous, i.e., intrinsically present in biological tissue or synthesized after introducing a precursor molecule, or exogenous, i.e., administrated as drugs) light-induced fluorescence spectroscopy can be classified either as autofluorescence spectroscopy or as induced fluorescence spectroscopy, respectively. The information on fluorescence lifetimes may be obtained using instruments with pulsed mode illumination and time-resolved detection [43]. The direct links between endogenous fluorophores and certain morphological and functional properties of living matter lead to distinguishable autofluorescence emission peaks and give an opportunity to monitor the state of biological tissues in vivo.

There is experimental evidence that the intensity of autofluorescence of normal cervical tissue is altered by the precancerous modifications of cervical epithelium [31]. The differences in fluorescence spectra of normal and precancerous cervical tissue are explained by the concomitance of two phenomena linked with the CIN progression. An increase in number of metabolically active mitochondria in epithelial cells with CIN development leads to the increase of epithelial fluorescence, while stromal fluorescence drops because of a decrease in density of the collagen matrix adjacent to neoplastic epithelium [31,44]. The overexpression of matrix metalloproteinases (enzymes responsible for the degradation of collagen cross-links, which are the main source of collagen autofluorescence) was found to be an early sign of malignant transformation in cervical neoplasia [45].

**Figure 6:**
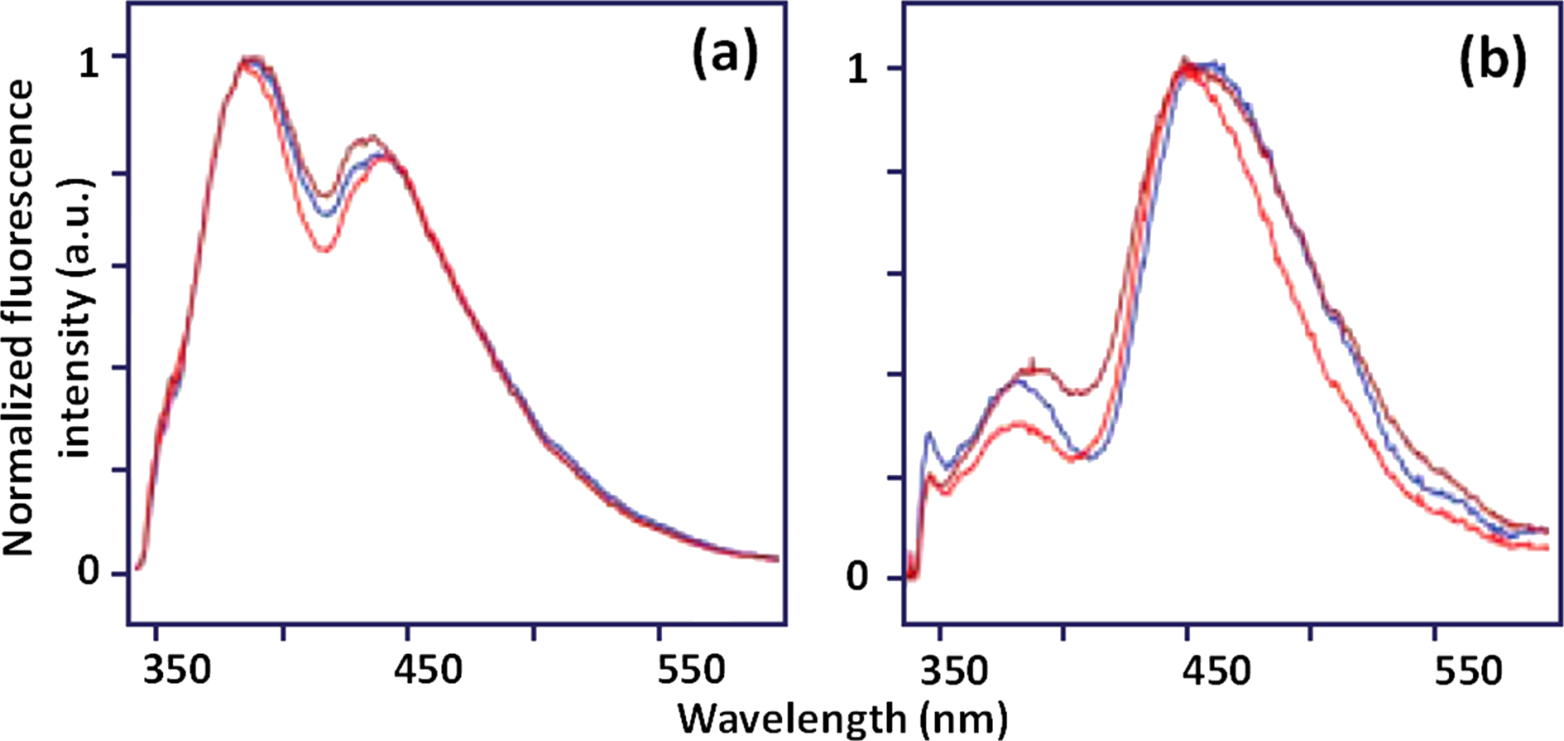
Site-to-site variations in fluorescence spectra measured at different pathologically confirmed (a) normal and (b) malignant tissue samples at 325 nm excitation wavelength. Reproduced with permission from [46], copyright 2006 Wiley-Liss, Inc.

Chidananda et al. [46] studied about 1000 autofluorescence spectra of cervical tissue specimens taken from 62 patients with different cervical pathologies. They reported a sensitivity and specificity of over 95% for CIN diagnostics using total fluorescence spectra resulting from the emission of individual endogenous fluorophores (e.g., collagen and the reduced form of nicotinamide adenine dinucleotide (NADH), the main tissue fluorophores in the visible wavelength range). The excitation wavelength was 325 nm. Principal component analysis (PCA) of the spectra and the intensity ratio of curve resolved fluorescence peaks was applied (Figure 6). Recent studies of autofluorescence spectra of biopsied specimens taken during colposcopy from 46 patients demonstrated both a significant decrease in collagen fluorescence (peak around 400 nm) and increase in NADH fluorescence (peak around 460 nm) in dysplastic tissues [47].

Combining information about the fluorescence of stromal collagen and epithelial NADH, Pandey et al. [47] reported a sensitivity of 96.5% for cervical neoplasia diagnosis. In vivo fluorescence spectroscopy studies reported the decrease of emission intensity combined with the shift of emission peak towards longer emission wavelengths for precancerous zones compared to healthy squamous tissue of the ectocervix.

Apart from the changes in cellular metabolic processes and in the extracellular tissue matrix induced by CIN progression, both scattering and absorption of light in tissues may significantly influence the measured fluorescence spectra modifying the intensity and width of specific peaks. Georgakoudi et al. [48] suggested combining the information from DRS and fluorescence spectra in order to remove the distortion of fluorescence spectra caused by tissue scattering and absorption and to determine the fluorescence spectra of NAD(P)H and collagen in vivo. The intrinsic (undistorted) fluorescence spectra from 35 patients taken at different (normal and abnormal) sites of the cervix during the colposcopy were analyzed. The results of these studies also showed that high-grade dysplastic lesions are characterized by low collagen fluorescence and high NADH fluorescence compared to non-dysplastic tissues.

Despite a clinically significant increase in NADH fluorescence and decrease in collagen fluorescence in the spectra measured on dysplastic cervical tissue the age of the patient may affect the fluorescence-based diagnosis of CIN. Some age-related changes of cervical tissue modify the fluorescence spectra in a similar way as dysplasia [47,49]. Due to a wide inter- and intra-patient variability of fluorescence spectra there is a need for the development of advanced mathematical algorithms for the analysis of fluorescence signals to provide the consistent and reproducible diagnosis of cervical neoplastic lesions [46,50]. The preprocessing (filtering, co-registration) of reflectance and fluorescence images, the reduction of image data by PCA, the image clustering by the K-means clustering algorithm and the use of the nonparametric K-nearest neighbor (KNN) classifier for the image segmentation was implemented by Milbourne et al. [51] for the diagnosis of high- and low-grade lesions of the cervix. The results of this pilot study in 46 patients showed that using an appropriate classifier on the multispectral digital colposcope data may produce algorithmic maps that correlate well with histopathologic mapping.

The accuracy of the detection of CIN lesions with spectral autofluorescence measurements depends on several factors including (i) changes in autofluorescence background, which may influence the quantum yield of fluorophore, (ii) inhomogeneities in the optical properties of tissue, (iii) alterations of the tissue architecture (e.g., variable thickness of epithelial layer), (iv) the spectral dependence of the absorption of light by non-fluorescent chromophores such as hemoglobin. Weingandt et al. [52] observed a similarity of autofluorescence response from zones of severe inflammation and of CIN. This made the diagnostics difficult and led to an increased number of false positive results.

Gu et al. [53] suggested using fluorescence-lifetime imaging microscopy (FLIM) on haematoxylin and eosin (H&E) stained histological cuts of cervical tissue and a neural network classifier for the automated diagnosis of CIN lesions. This technique can overcome the limitations of conventional fluorescence microscopy because FLIM results are insensitive to fluorophore concentration and excitation power of the laser.

The growth of tumor in mice, inoculated with highly tumorigenic TC-1 cells immortalized using HPV type 16 proteins, was studied as a model of cervical cancer by Bae and co-workers [54]. Using an optical imaging system they detected the enhancement of protoporphyrin IX (PpIX) autofluorescence in tumor regions. This endogenous protein tends to accumulate in tumor tissue, and may help in effective localization and visualization of tumor lesions by PpIX fluorescence imaging

An intrinsic problem of fluorescence spectroscopy is linked to the fact that both intensity and contrast of autofluorescence in tissue are quite low. Often the spectral difference between normal and pathological tissue can be enhanced by external administration of fluorophores or fluorophore precursors. The preferential accumulation of exogenous fluorophores in abnormal cells [55] results in contrast enhancement, which helps to detect and stage the lesions [56–57]. However, possible side effects and a low accumulation rate of exogenous fluorophores may impede the clinical use of the method.

### Raman spectroscopy

**Figure 7:**
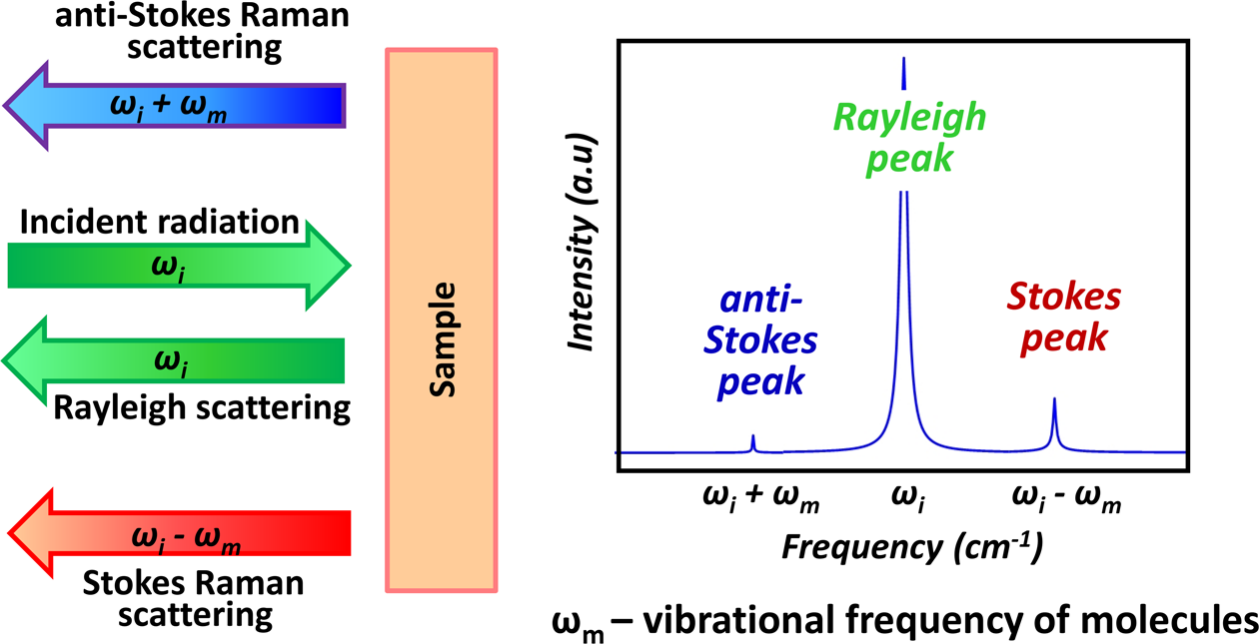
Raman vibrational spectroscopy for probing the molecular chemical bonds as well as crystal lattice vibrations. ω_i_ is the frequency of the incident radiation.

During the interaction of light with matter a number of different processes may take place: reflection, transmission, absorption, elastic and inelastic scattering of incident radiation. Raman spectroscopy (RS) is an optical technique that relies on inelastic scattering of light. The sample is usually illuminated with a monochromatic laser beam that vibrationally excites molecular chemical bonds. The energy of inelastically scattered light is changed by those vibrations that are strictly related to the structure of molecules. A plot of intensity of inelastically scattered radiation as a function of the difference of its frequency from the frequency of the incident radiation is called Raman spectrum. Consequently, positions, shapes and relative intensities of the peaks in a Raman spectrum carry valuable information about both chemical composition and morphology of the sample. That is why RS performs well as a versatile optical technique for chemical and structural characterization of studied samples in a rapid and non-destructive manner (Figure 7).

The biochemical components of tissue (e.g., proteins, lipids and carbohydrates) contribute to the measured Raman spectra by superposition of their individual Raman signals. The combinations of these components, which are specific for the different type and physiological status of tissue, produce a unique biochemical “signature” of the sample in the form of particular fingerprint-like spectral features in the Raman spectrum.

It suggests that Raman spectroscopy may be used as a tool to detect early biochemical changes at a molecular level that are associated with the precancerous modifications of tissue. During the last years the potential of RS as label-free diagnostics technique for the detection of different types of cancers has been studied by many research groups both in vivo and ex vivo [58–65].

In one study of 44 patients Raman spectra were acquired from 356 normal and 120 precancerous sites during the colposcopy in the fingerprint (FP, 800–1800 cm^−1^) and high wavenumber (HW, 2800–3700 cm^−1^) spectral regions [62]. Differences in Raman spectra of normal and dysplastic cervical tissue were observed at wavenumbers related to proteins, lipids, glycogen, nucleic acids, and the water in tissue. The multivariate statistical analysis yielded a sensitivity of 85.0% and a specificity of 81.7% using integrated FP/HW Raman spectroscopy for the in vivo diagnosis of cervical precancerous lesions.

Results of studies of 79 patients showed that in vivo Raman spectroscopy combined with logistic regression can differentiate HSIL zones from benign conditions with a similar sensitivity of 89% and a higher specificity of 81% compared to colposcopy in expert hands [66].

The use of Raman spectroscopy for histological analysis of cervical tissue cuts is discussed in [60] and [64]. The Raman spectral mapping of the unstained histological cuts was performed with the spatial resolution of 18 µm. The spectral Raman data set was evaluated by K-means cluster analysis (KMCA). The regions with similar spectral and hence biochemical properties were clustered on a generated pseudo-color map.

In the spectrum averaged over the pixels from stromal layer cluster the Raman peaks at 853, 921, 938 and 1245 cm^−1^ were assigned to collagen, which is the major component of this layer. The accumulation of glycogen in the mature squamous cells of intermediate and/or superficial layers manifested itself by peaks at 480, 849 and 938 cm^−1^ in the spectrum averaged over the pixels from corresponding clusters. The differentiation of stromal, basal and superficial layers on a pseudo-color map of normal squamous cervical tissue was clearly observed [64]. The KMCA of Raman spectral data from cervical tissue with HSIL demonstrated the loss of differentiation of layers. The classifier clustered HSIL regions with basal layer. It proposes that cells of both regions of cervical tissue share common biochemical profiles.

The obvious advantages of Raman spectroscopy include (i) no specific requirements for sample preparation, (ii) the possibility to use this technique with fiber optics for ex vivo and in vivo measurements, (iii) a high spatial resolution suitable for imaging of subcellular components.

Typically, Raman scattering produces a very weak signal (with a spontaneous inelastic scattering cross-section of about 10^−30^ cm^2^·sr^−1^). So, one of the main difficulties of RS consists in separating the contribution of the weak intensity of the inelastically scattered light from the strong intensity of the Rayleigh scattering signal. Current solution consists in using notch or edge optical filters to cut the contribution of the Raman probing wavelength. To avoid the interference of the Raman signal with fluorescence emission, special attention should be paid to the selection of the laser excitation wavelength.

The improvement of the signal-to-noise ratio can be achieved by using ultrashort-pulsed laser sources (stimulated Raman scattering (SRS) and coherent anti-Stokes Raman scattering (CARS)) or metal nanoparticles (surface-enhanced Raman scattering, SERS). However, these improvements often increase the time of measurements and the complexity/cost of the instrument, which may hinder clinical applications of Raman spectroscopy. In addition, the spread of diagnostically relevant peaks across the Raman spectra requires the development of efficient classifiers, which can fully explore rich spectral information for accurate and reliable diagnostics. One of the promising applications of RS can be the monitoring of the patients undergoing chemotherapy. A priori knowledge of administrated drugs will help to detect the new Raman peaks. There will be no need for point-by-point scanning. Hence, the time of measurements can be significantly reduced.

### High-resolution microscopy

The optical techniques for CIN diagnostics discussed so far focused on macroscopic imaging or spectral probing of tissue. It is known that CIN lesions are characterized by morphological changes, such as modified tissue architecture, increased size of cell nuclei and increased nuclear/cytoplasmic ratio. The assessment of these morphological changes is currently done through microscopic histological analysis of biopsies (gold-standard diagnostics). Screening and diagnostics can be significantly improved by the high-resolution optical imaging technologies that image subcellular structures in vivo, thus, replacing tissue removal, processing, and examination by pathologists [67].

In vivo confocal microscopy is an optical technology that can non-invasively reconstruct three-dimensional cell structures from successive microscopic images taken at different depths (around 300–400 µm) within a thick tissue (so called optical sectioning). A point illumination and a pinhole placed at the optically conjugate image plane in front of the detector isolate light reflected or fluorescent from a finite volume and block scattered and out-of-focus light. This increases optical resolution and image contrast compared to conventional optical microscopy. The sample plane is scanned by focused laser beam and confocal images are built up point-by-point. The fluorescence scanning confocal microscopy is typically used for imaging in the majority of biological applications [68–70]. The use of reflectance confocal microscopy for tissue imaging is limited, but sometimes it can provide additional information from the samples with significant spatial variation of refractive index [71–72]. It is worth to mention that optical sections are imaged in a focal plane tangential to the tissue surface. This is not a typical view seen by pathologists, because standard histological cuts are orthogonal to the tissue surface.

**Figure 8:**
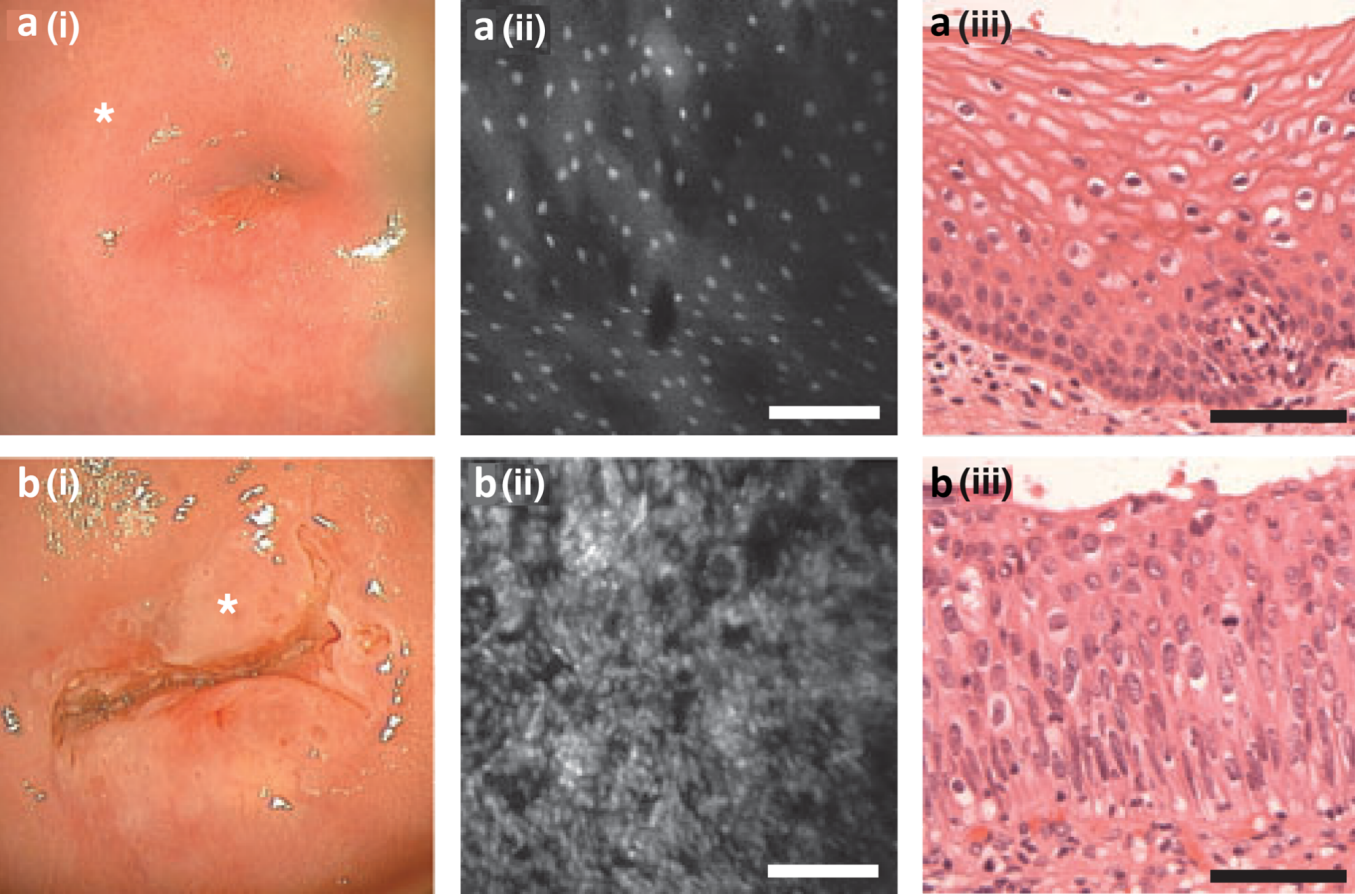
Cervical epithelium examined using (i) colposcopy, (ii) confocal endomicroscopy and (iii) conventional histology (H&E staining). (a) Normal cervix; (b) cervical CIN 3 lesion. *Confocal image site. Scale bars = 100 µm. Reproduced with permission from [76], copyright 2006 Wiley-Liss, Inc.

Confocal microscopy has been extensively used in different branches of medicine [69–72]. Due to its ability to provide real-time structural information on superficial layers of tissue this technique was also applied for the detection of precancerous lesions of the uterine cervix [73–76]. A fiber-optic reflectance confocal microscope was used by Carlson et al. [74] for in vivo imaging of cervix. They demonstrated an increase of nucleus-to-cytoplasm ratio with scanning depth in normal epithelium, but there was little change of this ratio from the upper layer to the basal layer in the images of dysplastic epithelium. Tan et al. [76] used fluorescence confocal endomicroscopy for in vivo microscopic imaging of cellular structures during colposcopy. Confocal imaging and histology of normal cervix tissue (Figure 8 a(ii), a(iii)) showed a uniform arrangement of epithelial cells through the full thickness of squamous epithelium.

CIN lesions were characterized by increased nuclear density and size, and the presence of atypical cells. Examination of a CIN 3 lesion with a confocal endomicroscopic imaging probe (site marked by the asterisk in the colposcopy image (Figure 8 b(i)) showed significant variation in nuclear size and shape (Figure 8 b(ii)). Histological analysis confirmed precancerous cell modifications over the full epithelial thickness (Figure 8 b(iii)). They reported a sensitivity of 97% for CIN detection, a specificity of 80% for predicting the grade of dysplasia for normal tissue to CIN 1 and 93% for CIN 2 to CIN 3 lesions.

A low-cost high-resolution microendoscope (HRME) was developed and used for the direct visualization of neoplastic biomarkers (increase in nucleus-to-cytoplasm ratio and nuclear density, pleomorphic nuclei) during colposcopy [77–79]. Grant et al. [79] performed HRME imaging by placing a fiber-optic probe tip in contact with colposcopically abnormal and normal sites. Before microendoscopic imaging a topical solution of proflavin (fluorescent DNA label that stains the nuclei and makes them appear brighter than the cytoplasm of the cell) was applied to the cervix. In pilot studies involving 59 women the HRME images were obtained from 84 colposcopically abnormal sites and 59 colposcopically normal sites. They reported a sensitivity of 92% and specificity of 77% for CIN 2+ detection using parameters calculated from HRME images of 59 abnormal sites (nucleus-to-cytoplasm ratio, mean nuclear area and median nuclear eccentricity). They acknowledged a lower specificity of HRME image-based diagnostics (67%) in their previous studies [77] where they used one parameter from HRME image (nucleus-to-cytoplasm ratio) alone for the diagnostics. The majority of the sites with false-positive diagnosis were affected by chronic inflammation.

A set of images from over 60 patients obtained by fluorescence confocal endomicroscopy was used for ex vivo and in vivo studies of four types of cervical tissue relevant for the diagnostics: normal columnar epithelium, normal and precancerous squamous epithelium, and stromal tissue [80]. Researchers acknowledged the challenge of reliable differentiation of all four types of cervical tissue by fluorescence endomicroscopy alone because of structural similarities of HSIL and stromal/columnar tissues in confocal endomicroscopic images. However, the capacity of confocal fluorescence microscopy to accurately discriminate between HSIL and LSIL/normal tissues at various imaging depths was confirmed [80–81].

### Nanotheranostics

**Figure 9:**
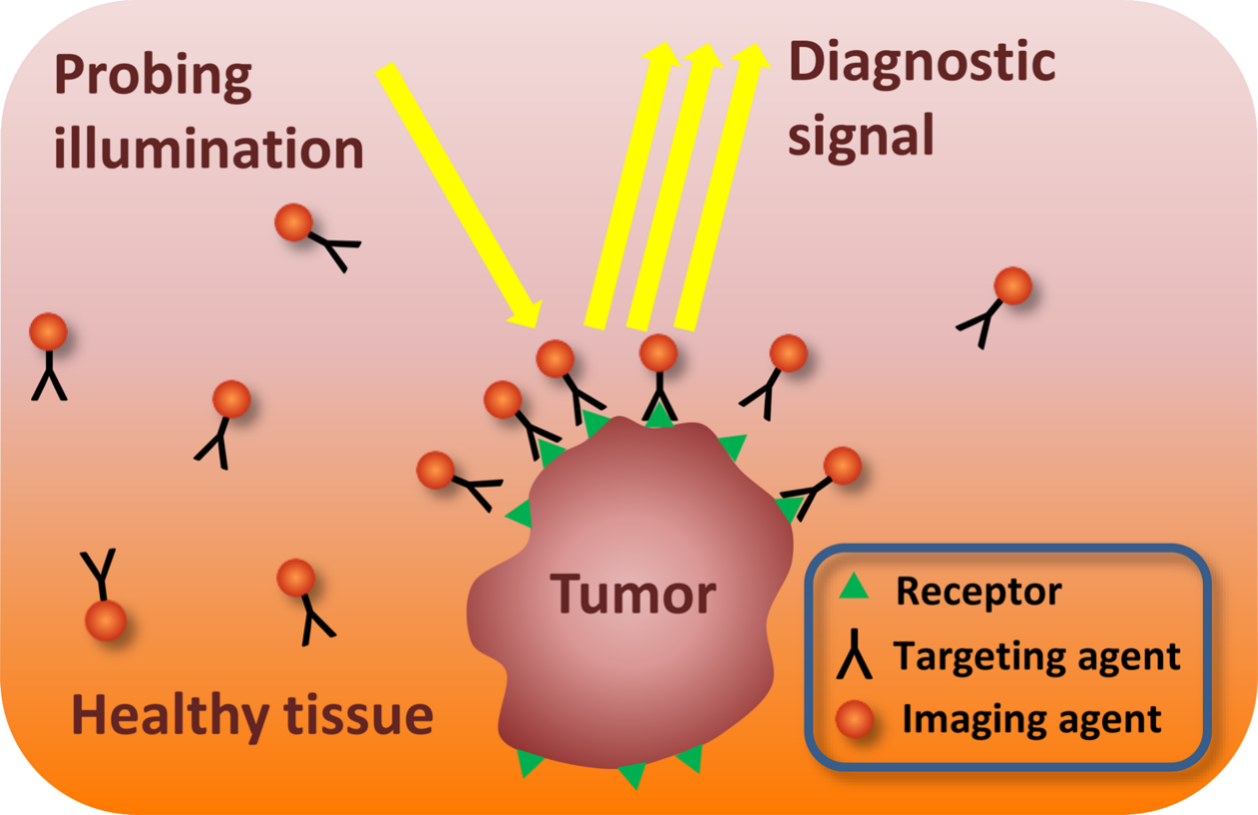
Illustration of optical molecular-targeted imaging with nanoparticles.

The rapid progress of nanotechnology had an important impact on cancer management research. The variety of new nanoscale platforms (gold nanoparticles, quantum dots, nanocages, carbon nanotubes) are used for cancer theranostics, which means the simultaneous diagnosis and treatment of diseases [82–86]. These nanoobjects can be used for a non-invasive monitoring of cellular processes at a molecular level. It has been confirmed that there is a strong interaction of nanoobjects with a size of less than 100 nm (i.e., which are much smaller than normal human cells) with biomolecules such as receptors, enzymes, and antibodies on the cell surface and inside the cell [87]. By surface coating, functionalization, and integration with different bioconjugated targeting agents those nanoparticles can be used for molecular-selective recognition of cancer biomarkers. The overexpression of specific biomarkers with cancer development will lead to the increase in concentration of optically active nanoobjects in the tumor zone and, consequently, to the diagnostic contrast enhancement (Figure 9).

It is known that progression of CIN from mild dysplasia to invasive cancer is accompanied by the increase in level of epidermal growth factor receptor (EGFR). The overexpression of EGFR has been correlated to uncontrolled cell growth and inhibition of cell apoptosis. Hence, EGFR can be used as a unique molecular tumor marker [88].

The contrast agents consisting of a targeting agent conjugated with optically active labels (metal nanoparticles, quantum dots) can be used for in vivo imaging of this biomarker. Sokolov et al [89] reported the use of gold nanoparticles for the molecular targeted imaging of the specific biomarker of cervical cancer. The bioconjugates of gold nanoparticles (approximately 12 nm in diameter) with antibodies against EGFR have been used to increase the contrast during in vitro confocal reflectance and confocal fluorescence imaging of normal and abnormal cervical cells. The high affinity of antibodies to EGFR and the overexpression of EGFR in tumor cells lead to the agglomeration of gold nanoparticles in tumor zone. The scattering cross-section per particle increases when particles agglomerate. It leads to a non-linear enhancement in scattering resulting in a large optical contrast between isolated gold particles and agglomerated gold particles in tumor tissue in both confocal reflectance and confocal fluorescence images of cervical tissue specimens [89].

In recent years many research groups explored the potential of using quantum dots (QDs) as inorganic fluorophores for cellular imaging [57,83,85,90–93]. The unique optical properties of semiconductor quantum dots including quantum confinement effect, wide absorption spectrum (i.e., broad excitation band), and narrow emission spectrum (i.e., tunable fluorescence emission via QD bandgap engineering) combined with low toxicity and resistance to photo bleaching [93] make them ideal candidates for multi-wavelength cellular imaging. Because of the small size QDs can be effectively used for labeling molecular targets at both cellular and subcellular levels. Despite the above mentioned advantages of using QDs for molecular imaging in cancer theranostics the possible side effects (toxicity, disruption of cellular processes) also need to be considered [90].

### Mueller polarimetry

There is an emerging set of optical techniques based on the detection of changes in the polarization of light instead of (or together with) intensity measurements. Apart from the intensity and wavelength of probing light its polarization can carry important information about the sample. Many research groups work in the field of biomedical applications of polarized light [94–103].

Even the simplest orthogonal state contrast (OSC) polarimetric techniques provide data about the polarimetric characteristics of the sample. Typically, the sample is illuminated with linearly or circularly polarized white light. Two set of measurements are performed, detecting the intensity of signal after interaction with sample through another linear or circular polarizer, set parallely (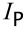) and orthogonally (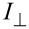) to the polarization state of the illuminating light. The OSC parameter is then calculated from these two measurements as 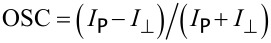. The diagnostic utility of this optical technique relies on the fact that polarized light loses its polarization when undergoing multiple scattering events within biological tissue. The part of backscattered light that preserves its polarization was most probably scattered only once or reflected at the sample surface. Thus, the differential signal removes the contribution of light that has been diffused deeply within tissue and keeps the contribution of the superficial layer at which epithelial cancer starts.

The studies of spectra or images of OSC polarimetric measurements for the detection of colon cancerous polyps [94], skin cancer [95], and cervical precancerous lesions [104–105] revealed the enhancement of contrast between normal and pathological zones of tissue. Balas et al. [106] reported on using 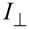 measurements for eliminating the surface reflectance component during time-resolved imaging of the whitening of cervical neoplasia after the application of acetic acid.

The OSC techniques make use of only two out of four components of the Stokes vector


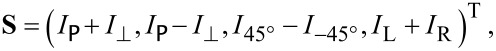


where *I*_45°_ and *I*_−45°_ denote the intensities which would be measured through ideal linear polarizers oriented along either +45° or −45°, respectively, in the plane perpendicular to the direction of light propagation and *I*_L_ and *I*_R_ are the intensities transmitted by left-handed or right-handed circular polarizers, respectively [107]. The linear transformation of the Stokes vector of incident light **S^i^** interacting with a sample is described by the matrix equation **S^o^** = **MS^i^**, where **M** is the 4 × 4 real Mueller matrix of the sample. This matrix provides the most complete description of the polarimetric response of any medium (even partially or fully depolarizing) to the illumination with polarized light in the absence of non-linear effects. So, using the Stokes–Mueller formalism has proven to be necessary when dealing with biological samples. Rich polarimetric information about the sample properties is contained in the coefficients of the Mueller matrix. Currently, the phenomenological approach based on polar decomposition of the Mueller matrix by the Lu–Chipman algorithm [108] is widely accepted by many research groups [97,102–103,105,109–110] for the interpretation of basic polarimetric properties of the sample. The measured Mueller matrix **M** is decomposed into the product of three matrices: **M = M_Δ_M_R_M_D_**, where **M_Δ_**, **M_R_**, and **M_D_** are the Mueller matrices of depolarizer, retarder and diattenuator, respectively. Finally, the scalar values of depolarization, retardance and diattenuation, as well as the orientation of the optical axes of the retarder and the diattenuator can be obtained from the matrices **M_Δ_**, **M_R_**, and **M_D_**. Strictly speaking, these parameters represent a set of “effective” optical markers of tissue. Lu–Chipman decomposition implies a sequential order of elementary polarimetric properties along the trajectory of the probing beam, whereas these polarimetric properties can be mixed within the volume of tissue. Nevertheless, these effective values of depolarization and retardance are found to be the important parameters for the polarimetric analysis.

Shukla et al. [109] obtained polarimetric images of histological slides of cervical tissue by applying Lu–Chipman decomposition of experimental Mueller matrices and analyzed them in order to discriminate normal tissue and CIN lesions. They found that values of scalar retardance drop in stromal areas adjacent to neoplastic epithelium. It can be explained by the structural reorganization of the extra-cellular collagen matrix accompanying early precancerous modifications of the epithelium [31–32]. The observed increase in depolarization power in neoplastic epithelial zones of tissue was attributed to an increasing scattering coefficient due to the increase of cell density.

**Figure 10:**
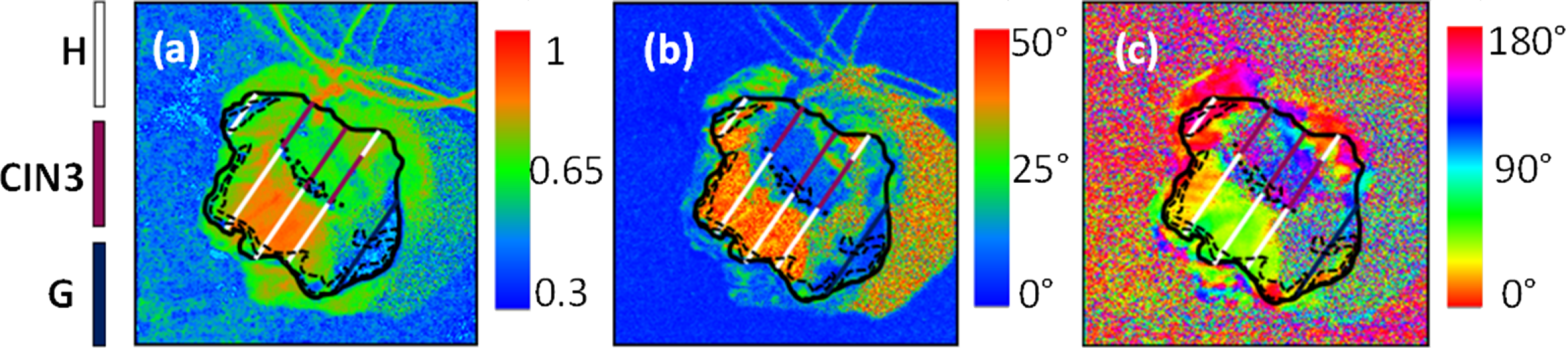
Polarimetric images of a cervical specimen taken at 550 nm: (a) depolarization (b) scalar retardance and (c) azimuth of optical axis. The colored lines show the position of cuts and results of histological analysis (white: healthy squamous epithelium (H), violet: CIN 3, black: glandular epithelium (G)). Reproduced with permission from [105], copyright 2013 Optical Society of America.

During in vivo clinical studies linear OSC images of healthy uterine cervices acquired during colposcopy demonstrated a strong change of OSC contrasts with a 90° periodicity (i.e., a strong optical anisotropy) when the azimuth of the polarizer was varied [105]. Contrary to that the OSC contrasts in CIN zones, confirmed by following histological analysis of corresponding biopsies, showed no dependence on the azimuth variation. Consequently, CIN zones behaved as an isotropic depolarizer. The ex vivo polarimetric images of scalar retardance and depolarization power calculated from the Mueller matrix of a fresh cervical specimen measured at 550 nm are shown in Figure 10. None of studied cervical specimens demonstrated noticeable diattenuation. Healthy regions of cervix covered with squamous epithelium exhibited strong birefringence (optical index anisotropy), which vanished in precancerous regions even for LSIL. The orientation of the optical axis of retarder became completely random in CIN zones.

This effect was attributed to the degeneration of stromal collagen beneath the precancerous epithelial lesions [31–32]. The depolarization power is found to decrease monotonously with precancerous evolution. Combining both scalar retardance and depolarization power values it is possible to delimit the zone of benign modifications of cervical tissue (Figure 10a,b).

At first glance the trends in depolarization look contradictory to the results of Shukla et al. [109]. However, the imaging plane of a Mueller polarimeter is orthogonal to the plane of histological cuts of tissue seen by pathologists. Moreover, the images of thick tissue specimens were taken in backscattering configuration [105] compared to Mueller polarimetric transmission measurements of thin histological cuts by Shukla and co-workers [109]. It suggests that “effective” optical polarimetric biomarkers of tissue (scalar retardance, azimuthal angle of retarder optical axis and depolarization) extracted from in vivo Mueller polarimetric images in clinical settings will be sensitive not to precancerous epithelial transformations but rather to stromal modifications induced by CIN [111]. Hence, the decrease of depolarization power in CIN zones of thick tissue can be attributed to both a decrease of light scattering and an increase of absorption [112] due to reorganization of the collagen matrix and stromal angiogenesis [28–30].

**Figure 11:**
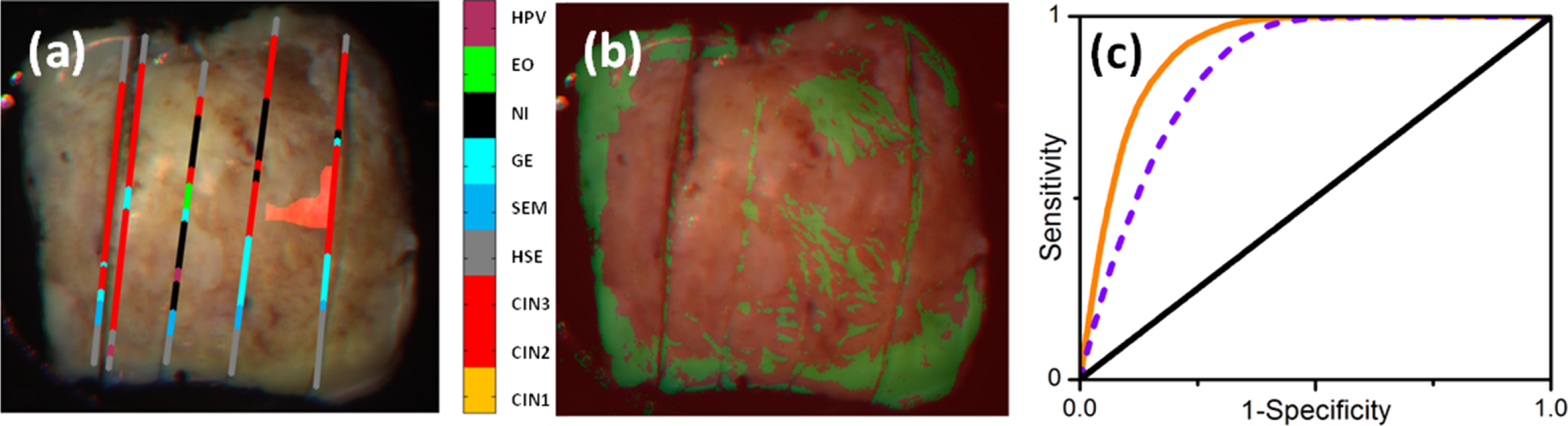
(a) Histological map (colored lines) superimposed on an RGB image of a conization sample; HPV: epithelium infected by HPV; EO: external os of cervix; NI: non-identified epithelial zones, GE: glandular epithelium; SEM: squamous epithelium metaplasia; HSE: healthy squamous epithelium; CIN 1–3: squamous intraepithelial neoplasia of grade 1–3; (b) diagnostic (red: CIN 2–3, green: all other conditions) image segmentation using a threshold of 10.1° for the value of scalar retardance *R* for measurements performed at 450 nm; (c) receiver operating characteristic (ROC) curves (violet dashed: diagnostics based on scalar retardance values only, orange: diagnostics based on combination of scalar retardance and depolarization power values). Images adapted from [113], copyright 2016 Society of Photo-optical Instrumentation Engineers.

Ex vivo studies of 17 fixed cervical specimens performed with a multi-spectral Muller imaging polarimeter [113] showed optimized values of sensitivity and specificity of about 83% for HSIL diagnosis when using both scalar retardance and depolarization power values as decision variables and histological analysis of pathologists as gold-standard diagnostics (Figure 11).

This suggests that Mueller polarimetry as wide-field imaging technique can greatly enhance colposcopy performance for the detection of CIN zones provided the raw data are processed by properly chosen algorithms. Finally, since polarimetric imaging is sensitive to the overall conditions of the collagen in the extracellular matrix (e.g., spatial organization, density and fiber length), it may also be relevant for the optical diagnostic of various gynecological pathologies involving connective tissues (e.g., preterm birth [114] and female genital prolapse [115–116]).

### Optical coherence tomography

Optical coherence tomography (OCT) is an optical technique for non-invasive cross-sectional imaging of biological tissue. This technique makes use of low-coherence interferometry with a near-infrared light source to create two-dimensional images of tissue cross-sections by exploring elastic light scattering from internal tissue microstructures [117]. OCT provides depth-resolved images, where the contrast results from the spatial difference in refractive indices of layers and structures within the tissue. The high resolution of OCT (2–20 µm) and a depth of penetration up to 2 mm allow clinicians to visualize the sub-surface tissue in real time at a spatial resolution better than that available with other optical diagnostics techniques. The depth resolution of OCT is decoupled from its transverse resolution.

Nowadays OCT is the reference technique in ophthalmology [118–121] and has been clinically tested in dermatology, otolaryngology and gastroenterology [122–125]. Recently OCT has been shown to be an efficient adjunct to colposcopy for the management of cervical neoplasia [126–128]. Because the resolution of OCT approaches the cellular tissue level, this optical technique demonstrated its potential for guiding biopsies during colposcopy and for monitoring CIN treatment [129].

Normal squamous cervical tissue exhibits a well-organized three-layer architecture [126]. Prior studies revealed that the lack of this specific structure in OCT images of squamous cervical tissue can be used as a fingerprint of malignancy, which allows for discriminating “benign” and “malignant” OCT images [129–131].

**Figure 12:**
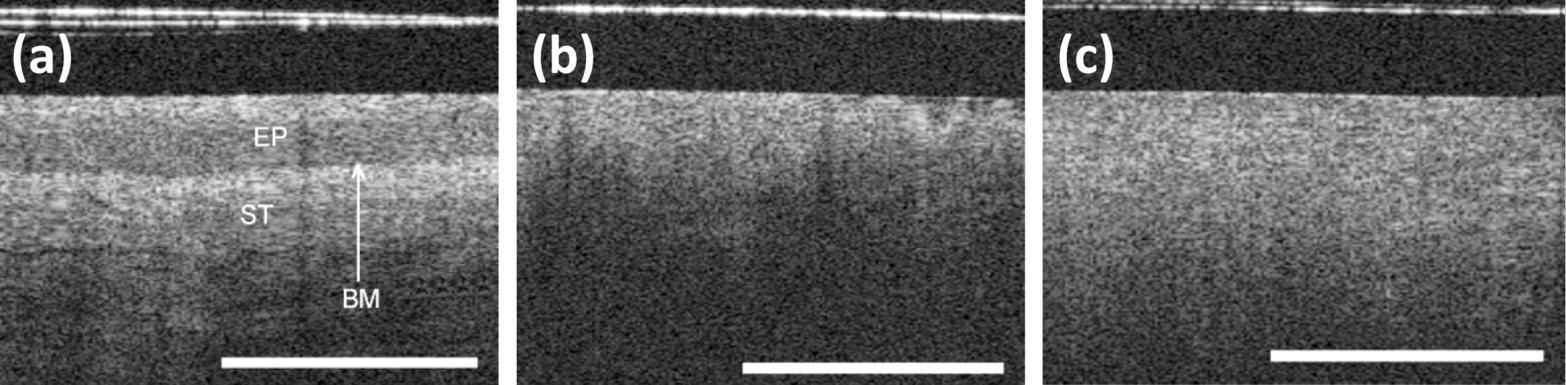
(a) OCT image of normal cervical tissue (BM: basement membrane, EP: epithelium, ST: stroma); (b) OCT image of a CIN 3 lesion; (c) OCT image of invasive carcinoma (length of white bar: 1 mm). Reproduced with permission from [131], copyright 2010 ISUOG.

In the OCT image of healthy squamous cervical tissue a basement membrane (BM) is not resolved because of the lack of OCT resolution (Figure 12a). However, a sharp interface between the epithelium and stroma is clearly seen on OCT image. Both HSIL and invasive carcinoma are characterized by loss of layered tissue architecture and an increase in tissue microstructural disorder in OCT images (Figure 12 b,c). The stromal layer demonstrates columnar proliferation towards the surface of tissue in the OCT image of a CIN 3 lesion (Figure 12b). The invasive carcinoma manifests itself in the OCT image as unstructured homogeneous highly backscattering region with a complete loss of layered tissue architecture (Figure 12c). The basement membrane is broken and the microstructure of the tissue is no longer preserved.

Studies on using OCT for the detection of CIN and invasive carcinomas were carried by Gallwas and co-workers [131]. During the colposcopy in 60 women 610 OCT images were acquired from colposcopically abnormal and normal zones of the ectocervix. OCT images were independently evaluated by two experts and then matched to histological diagnoses of the corresponding biopsies. A sensitivity of 95% and a specificity of 46% for the detection of precancerous (CIN) and cancerous lesions were reported for OCT technique. A similar study in 120 women was performed for the evaluation of the accuracy and reproducibility of OCT diagnostics for both detection of CIN lesions and identification of CIN grades [126]. With the threshold at CIN 1 the sensitivity varied between 98% and 96%. Defining the threshold at CIN 2 the sensitivity calculated for both experts was 86% and 84%, respectively. A specificity of 39–41% was reported with the threshold at CIN 1. The specificity increased to 60–64% when the threshold was defined at CIN 2. These studies prove that OCT is highly sensitive in identifying precancerous lesions and invasive cancer of the uterine cervix. The relatively low specificity of OCT was attributed to the difficulties in distinguishing the OCT images of mild dysplasia (CIN 1) and tissue inflammation/benign modifications. It was shown that the mean brightness of the cervical epithelium layer in OCT images of squamous cervical tissue has the potential to become an optical marker for the differentiation between normal tissue, LSIL, HSIL and invasive cancer [132].

A study with 299 women on using OCT as adjunct to colposcopy for improving its sensitivity and specificity in a real-time clinical evaluation were conducted by Liu and co-workers [133]. They demonstrated that the specificity increased from 83% to 93% by adding OCT to colposcopy, but the sensitivity for CIN 2+ lesions decreased.

Gallwas et al. [134] suggested combining an OCT device with a microscope for the detection of CIN lesions. In that study 160 OCT images of excised cervical specimens were taken under microscopic guidance. The OCT images were independently analyzed by two experts and later compared to the histological gold-standard diagnosis, resulting in a sensitivity of 88% (second investigator 84%) and a specificity of 69% (65%) in detecting HSIL. They expect that the integration of an OCT instrument into the colposcope may be beneficial compared to scanning with the OCT probe and may improve the accuracy of colposcopic examinations of the cervix.

Polarization-sensitive OCT (PS-OCT) combines the spatial information on the polarization state of light scattered from tissue with the recorded intensity of interference fringes [135]. Depth-resolved images of Stokes vector parameters allow for the determination of the degree of polarization and the orientation of the optical axis in anisotropic turbid media, thus providing additional contrast in cross-sectional OCT images of the sample.

Lee et al. [136] examined cervical conization specimens from 18 patients with PS-OCT (71 images were taken) for the detection of CIN lesions. It was demonstrated that PS-OCT can improve the specificity of diagnosis when interpreting “difficult” OCT intensity images. From the images of the degree of circular depolarization DOCP = *S*_3_/*S*_0_, the slope of axial decay of DOCP signal near the cervical epithelium was determined by a linear fitting procedure. Using the abovementioned slope as parameter for CIN diagnostics a sensitivity of 94.7% and a specificity of 71.2% was obtained for a slope threshold value of 1.8 mm^−1^.

### Combined techniques

Several studies reported using the combination of different optical (and non-optical) techniques to improve the performance of CIN diagnostics. Georgakoudi et al. [137] developed a trimodal spectroscopy (TMS) combining intrinsic fluorescence, diffuse reflectance, and light scattering spectroscopy for the detection and analysis of CIN lesions. During colposcopic examination a white light reflectance spectrum and autofluorescence spectra at ten excitation wavelengths were acquired using a flexible optical contact probe. Light scattering spectra were obtained by subtracting the contribution of diffuse reflectance from the measured reflectance. Intrinsic fluorescence spectra provided information about the metabolic state of the epithelium and adjacent stromal collagen. Information about scattering and absorption properties of epithelium and stroma was extracted from light scattering and diffuse reflectance spectra, respectively. Data collected from 44 patients demonstrated that the sensitivity and specificity of TMS was higher compared to the results of each individual technique alone.

Freeberg et al. [33] reported the results of a screening trial comprising 1000 patients and a diagnostic trail comprising 850 patients with combined fluorescence and reflectance spectroscopy using a fiber-optic probe for detecting cervical neoplasia. According to their analysis there is a distinguishable difference in mean intensity values measured on normal cervical tissue and HSIL. However, type of tissue (squamous or columnar) and patient age were confounding factors for the performance of combined fluorescence and reflectance spectroscopy diagnostics. The clinical trials on 227 patients with an optical detection system (ODS; combining scanning fluorescence spectroscopy, diffuse reflectance spectroscopy and video imaging) as an adjunct to colposcopy showed statistically significant improvement in the detection of histologically confirmed CIN 2–3 lesions compared to conventional colposcopy [138].

Weber et al. [139] measured in vivo reflectance and fluorescence spectra of normal and precancerous cervical tissue in 330 patients using a fiber-optic point-probe. By means of an analytical model they extracted diagnostically relevant parameters from the spectral data. They reported a sensitivity of 85% and a specificity of 51% of their technique relative to the gold standard of histopathology analysis.

A multimodal hyperspectroscopy (MHS) instrument that combines fluorescence and reflectance spectroscopy was tested in 1607 women at risk for cervical dysplasia [140]. The sensitivity of MHS for CIN 2+ lesions was 91.3%. The specificity was 38.9% for women with normal or benign histology and 30.3% for women with CIN 1 histology.

The comparative studies of diffuse reflectance and Raman spectroscopic measurements performed in vivo with a fiber-optic probe on 22 patients (67 tumor spectra and 22 normal cervix spectra) showed a slightly better diagnostic accuracy of Raman spectroscopy [141]. The sensitivity and specificity of RS were estimated as 91% and 96%, respectively, compared to a sensitivity of 85% and a specificity of 95% for DRS diagnostics. Some inherent features of Raman systems (price, complexity and dimensions) suggest using them in stationary settings, while compactness, portability and low cost of DRS systems can make them an instrument of choice for field applications.

A hybrid optical imaging modality that explores photoacoustic effect was used by Peng et al. [142] for the detection and grading of precancerous and cancerous lesions of the cervix (in vitro studies). The technique is based on the absorption of light by tissue, which creates a temperature gradient, and the associated raise of pressure, which generates ultrasonic waves. Acoustic detectors receive these waves and provide the signals to generate images. Optical absorption in tissue is mainly due to hemoglobin; hence, photoacoustic imaging provides an enhanced image contrast for vascular system, hemodynamics and oxygen metabolism, which all can be used as biomarkers for the detection of tissue malignancy. Using ultrasound as a response signal allows for a deeper penetration depth compared to pure optical imaging systems. It suggests photoacoustic imaging for the detection of lesions in the endocervix, which is not accessible for the direct observation under colposcopy [142].

The efficacy and advantages/disadvantages of new alternative technologies or technologies adjunct to colposcopy using multimodal hyperspectroscopy, dynamic spectral imaging, OCT, confocal microcolposcopy, electrical impedance spectroscopy and combined optical/electrical instruments are discussed in [143–147].

The use of all functionalities of smartphones for the wide-field imaging of the uterine cervix with white and green light sources and magnification lens for an enhanced visualization is suggested and being tested by MobileODT [148]. Another mobile battery-powered colposcope Gynocular by Gynius AB [149] was clinically tested for cervical examination in Sweden, Bangladesh, India and Uganda. These new instruments allow a medical practitioner to get relevant information on the spot, thus, making screening, diagnostics and treatment more effective and less expensive. Hence, the emerging techniques may also contribute to the reliable “screen-and-treat” cervical cancer programs in low- and middle-income countries wherever the Pap and/or HPV tests would be difficult to implement [150–151].

### Conclusion

Current programs for cervical cancer screening still rely on Pap/HPV tests for the primary screening and on colposcopy for the diagnostics and guiding biopsies, if necessary. With more countries introducing HPV vaccination programs the prevalence of HSIL is expected to drop. The challenges for standard colposcopy will grow, since correct diagnosis depends to a large extent on the experience of the operator trained to recognize the high-grade cervical dysplasia on a sheer number of cases.

To maintain the satisfactory level of sensitivity and specificity of HSIL diagnostics we need to improve the instruments and advance the screening procedures. The innovative biomedical optical imaging and spectroscopic techniques provide clinicians the possibility to inspect the epithelial volume and underlying tissue non-invasively and to extract an accurate information regarding tissue morphological and biochemical states. The choice of the most appropriate optical technique will always involve a trade-off between the technical parameters (such as spatial and spectral resolution, acquisition time and field of view) and medical diagnostic outcome (specificity and sensitivity) that can be achieved.

Currently, new optical instruments and contrast enhancement techniques for the accurate and reliable diagnostics of cervical neoplasia are still at an exploratory stage and have not yet been widely accepted for routine screening and diagnostics. Commercially available instruments are being tested by medical practitioners in real-life settings, while other devices still undergo clinical trials for the confirmation and optimization of their diagnostic performance.

Modern trends in biomedical optical instrumentation require the development of portable and cost-effective versions of medical devices. In particular, these needs are driven by the necessity to support global transition, namely, to deploy and use these instruments in low-resource countries. This is the problem of paramount importance for the screening, diagnosis and treatment of cervical cancer. Ideally the wide-field imaging (polarimetric or fluorescent) should be combined with optical point-probe measurements (e.g., Raman spectroscopy, confocal microscopy, OCT) and different contrast-enhancing techniques to perform the optical biopsy of tissue. Such instruments might be considered for the first-line screening and triage by optical means during the same medical visit, thus, significantly reducing the cost of cervical cancer prevention programs.
